# Item

**Published:** 1910-09

**Authors:** 


### ITEM

Battle & Co., of St. Louis, have just issued No. 13 of their series of charts on dislocations. This series forms a most valuable and interesting addition to any physician's library. They will be sent free of charge on application, and back numbers will also be supplied. If you have missed any of these numbers, write Battle & Co. for them before the supply is exhausted.

